# Predictors of left ventricular remodeling after ST-elevation myocardial infarction

**DOI:** 10.1007/s10554-017-1131-1

**Published:** 2017-04-07

**Authors:** Tom Hendriks, Minke H. T. Hartman, Pieter J. J. Vlaar, Niek H. J. Prakken, Yldau M. Y. van der Ende, Chris P. H. Lexis, Dirk J. van Veldhuisen, Iwan C. C. van der Horst, Erik Lipsic, Robin Nijveldt, Pim van der Harst

**Affiliations:** 1 Department of Cardiology, University of Groningen, University Medical Center Groningen, Hanzeplein 1, 9700 RB Groningen, The Netherlands; 2 Department of Radiology, University of Groningen, University Medical Center Groningen, Groningen, The Netherlands; 3 Department of Critical Care, University of Groningen, University Medical Center Groningen, Groningen, The Netherlands; 4 Department of Cardiology, VU University Medical Center, Amsterdam, The Netherlands

**Keywords:** Magnetic resonance imaging, Myocardial infarction, Left ventricular remodeling, Multivariable analysis

## Abstract

Adverse left ventricular (LV) remodeling after acute ST-elevation myocardial infarction (STEMI) is associated with morbidity and mortality. We studied clinical, biochemical and angiographic determinants of LV end diastolic volume index (LVEDVi), end systolic volume index (LVESVi) and mass index (LVMi) as global LV remodeling parameters 4 months after STEMI, as well as end diastolic wall thickness (EDWT) and end systolic wall thickness (ESWT) of the non-infarcted myocardium, as compensatory remote LV remodeling parameters. Data was collected in 271 patients participating in the GIPS-III trial, presenting with a first STEMI. Laboratory measures were collected at baseline, 2 weeks, and 6–8 weeks. Cardiovascular magnetic resonance imaging (CMR) was performed 4 months after STEMI. Linear regression analyses were performed to determine predictors. At baseline, patients were 21% female, median age was 58 years. At 4 months, mean LV ejection fraction (LVEF) was 54 ± 9%, mean infarct size was 9.0 ± 7.9% of LVM. Strongest univariate predictors (all p < 0.001) were peak Troponin T for LVEDVi (R^2^ = 0.26), peak CK-MB for LVESVi (R^2^ = 0.41), NT-proBNP at 2 weeks for LVMi (R^2^ = 0.24), body surface area for EDWT (R^2^ = 0.32), and weight for ESWT (R^2^ = 0.29). After multivariable analysis, cardiac biomarkers remained the strongest predictors of LVMi, LVEDVi and LVESVi. NT-proBNP but none of the acute cardiac injury biomarkers were associated with remote LV wall thickness. Our analyses illustrate the value of cardiac specific biochemical biomarkers in predicting global LV remodeling after STEMI. We found no evidence for a hypertrophic response of the non-infarcted myocardium.

## Introduction

Myocardial injury caused by ST-elevation myocardial infarction (STEMI) leads to left ventricular (LV) remodeling, resulting in functional and structural LV changes [1]. Over time, remodeling adversely affects cardiac function and can lead to significant morbidity and mortality [2]. To attenuate LV remodeling, early risk stratification is needed to allow monitoring and more aggressive treatment of high-risk patients.

The most widely investigated functional LV characteristic to predict patient outcome after STEMI is LV ejection fraction (LVEF) [3]. Several structural LV characteristics have also shown to be important predictors of cardiovascular adverse events and death, including LV end diastolic volume (LVEDV), end systolic volume (LVESV) and mass (LVM) [4–8]. Cardiovascular magnetic resonance (CMR) imaging is the current reference standard for assessing ventricular volumes and mass [9]. There is limited knowledge of the predictive value of clinical, biochemical and angiographic factors for LVM, LVEDV or LVESV after STEMI. The compensatory hypertrophic response of the remote non-infarcted myocardium (end diastolic wall thickness (EDWT) and end systolic wall thickness (ESWT)) might also play an important role in the remodeling after myocardial infarction but this has not been studied in humans.

The objective of this study is to increase our understanding of the determinants of LV structural remodeling after STEMI. We performed a retrospective analysis of the GIPS-III trial, in which CMR was performed 4 months after STEMI. The primary endpoint of the GIPS-III trial on LVEF has been published previously [10]. We studied the clinical, biochemical and angiographic determinants of LVEDV, LVESV and LVM indexed to body surface area (BSA), as global LV remodeling parameters, as well as EDWT and ESWT of the non-infarcted myocardium, as compensatory remote LV remodeling parameters.

## Materials and methods

### Study population and procedures

Data was used from all patients included in the GIPS-III trial who underwent CMR assessment. The GIPS-III trial was a single-center, randomized, placebo-controlled trial, including STEMI patients treated with percutaneous coronary intervention (PCI) at the University Medical Center Groningen between January 1, 2011 and May 26, 2013, previously described in more detail [10, 11]. Patients (N = 380) without known diabetes mellitus or previous myocardial infarction were randomized to take either placebo or a dosage of 500 mg metformin twice daily during a period of 4 months, in addition to standard protocolized care according to ESC guidelines [12]. The primary objective was to assess the effect of metformin on cardiac systolic function (LVEF), as measured with CMR at 4 months after STEMI. Major exclusion criteria were contraindications for CMR, the need for coronary artery bypass graft surgery, previous myocardial infarction, known diabetes, and severe renal dysfunction. Informed consent was obtained from all individual participants. All significant bystander lesions were treated by PCI before CMR assessment. In total, 271 CMR assessments were analyzed in a core laboratory.

### Cardiovascular magnetic resonance imaging

Cardiovascular magnetic resonance imaging (CMR) was performed 4 months after STEMI. LVEDV, LVESV, LVM, EDWT and ESWT were measured in addition to infarct size and LVEF, the primary outcome measure of the GIPS-III study [13]. Imaging was performed on a 3.0 T whole-body MRI scanner (Achieva; Philips) using a phased array cardiac receiver coil. During repeated breath holds, electrocardiogram-gated steady state free precession cine images were acquired in contiguous short-axis slices of 1 cm covering the entire LV. Endocardial and epicardial borders were outlined in the end systolic and end diastolic images. LVESV, LVEDV and LVM were calculated using the summation of slice method; LVM was measured at end-diastole. ESWT, EDWT and fraction of hyper-enhancement as measured with late gadolinium enhancement (LGE) were determined for each myocardial segment in a 16-segment model, based on the standard 17-segment model without the apical (17th) segment [14]. Infarct size was determined by summation of the volume of hyper-enhancement per slice, and expressed as percentage of total LVM. EDWT and ESWT of remote non-infarcted myocardium was defined as the mean EDWT and ESWT of all myocardial segments without LGE hyperenhancement. LVEDV, LVESV and LVM were indexed (i) to body surface area (BSA) according to the DuBois method [15]. Weight measurements at 4 months were used for indexation. An independent core laboratory (Image Analysis Center, VU University Medical Center, Amsterdam, the Netherlands) assessed the CMR scans, blinded for patient data.

### Laboratory analysis

Laboratory analysis was performed at the laboratory department of the University Medical Center Groningen. Blood samples for assessment of myocardial injury with creatine kinase (CK), myocardial band of CK (CK-MB), and Troponin T were collected according to study protocol at baseline, and at 3, 6, 9, 12, 18, 24, and 36 h after PCI. Using the trapezoid method, area under the curve (AUC) values were determined over the first 36 h post-PCI from a median of seven blood samples [16]. Other biochemical measures collected at baseline, at 2 weeks and at 6–8 weeks included N-terminal pro brain natriuretic peptide (NT-proBNP), glucose and estimated glomerular filtration rate (eGFR) as determined by the CKD-EPI formula [17].

### Statistical analysis

Pearson product-moment correlation coefficient (Pearson’s rho) was derived to assess correlation between infarct size and the investigated structural LV characteristics. The percentage of deviating values of LVMi, LVEDVi and LVESVi was derived by comparison with 95th percentile (P95) values in a healthy reference population [18]. Univariate and multivariable analyses were performed using linear regression. Results from univariariate analyses were presented with standardized beta (Std. *β*) and significance level (p-value). Age, sex and variables with p-value ≤0.10 after correction for age and sex (and BSA in EDWT and ESWT), were included in multivariable analysis. Collinear variables were removed from multivariable analysis, based on the lowest R^2^ when combined with age and sex, until variance inflation factors were lower than ten. Multivariable linear regression analyses were performed using a forward stepwise algorithm, with cutoff for entry set at a significance level 0.05 to find independent predictors of LVMi, LVEDVi, LVESVi, EDWT and ESWT. To validate the models within the study cohort, a backward stepwise algorithm was used with cutoff for removal set at significance level 0.10, and significance level set at 0.05. Additionally, the bootstrap method was utilized, using 1000 repetitions for model selection, and the order of most prevalent variables was compared with the previous models. Significant variables in the multivariable model were reported with Std. *β* and p-value. The coefficient of determination (R^2^) was derived to assess the proportion of variance predicted by the multivariable linear regression model. Variables within the models were tested for significant interactions, in conjunction with age and sex. Analyses were performed with STATA/IC version 13.0 (StataCorp LP, College Station, Texas, USA).

## Results

### Baseline characteristics

Cardiovascular magnetic resonance imaging (CMR) data was available for 271 (71%) patients participating in the GIPS-III trial. Baseline and follow-up clinical, biochemical, and angiographic characteristics are presented in Table 1.

**Table 1:** Baseline and follow-up clinical, angiographic and biochemical characteristics

Characteristics	Median (IQR) or n (%), n = 271
Age, years	58 (49–66)
Sex, % female	58 (21.4)
Body mass index, kg/m²	26.6 (24.4–29.4)
Weight, kg	83 (75–95)
Height, cm	178 (170–184)
Race/ethnicity, % Caucasian	257 (94.8)
Hypertension, %	75 (27.7)
Dyslipidemia, %	168 (62.0)
Current smoking, %	139 (51.3)
Stroke, %	1 (0.4)
Previous PCI, %	4 (1.5)
Systolic blood pressure, mmHg	132 (119–146)
Diastolic blood pressure, mmHg	83 (74–95)
Heart rate, beats/min	74 (64–84)
Ischemia time, min	155 (105–240)
Single vessel disease, %	195 (72.0)
Infarct-related artery, %	
LAD	111 (40.9)
LCX	46 (17.0)
RCA	114 (42.1)
Infarct-related artery TIMI flow pre-PCI, %	
0	153 (56.5)
1	21 (7.8)
2	47 (17.3)
3	50 (18.4)
Infarct-related artery TIMI flow post-PCI, %	
2	17 (6.3)
3	254 (93.7)
Myocardial blush grade, %	
0	5 (1.9)
1	19 (7.0)
2	57 (21.2)
3	188 (69.9)
Peak CK-MB, U/L	163 (76–328)
AUC CK-MB, U/L s 10^−8^	0.103 (0.044–1.86)
Peak CK, U/L	1420 (680–3110)
AUC CK, U/L s 10^−8^	1.02 (0.45–2.16)
Peak Troponin T, ng/L	49 (22–146)
AUC Troponin T, ng/L s 10^−5^	1.35 (0.48–2.65)
NT-proBNP, ng/L	80 (38–188)
NT-proBNP at 2 weeks, ng/L	555 (212–1150)
NT-proBNP at 6–8 weeks, ng/L	287 (126–631)
eGFR, mL/min	96 (86–103)
Glucose, mmol/L	8.2 (6.9–9.5)
HbA1c, %	5.8 (5.6–6.0)

*IQR* interquartile range, *PCI* percutaneous coronary intervention, *LAD* left anterior descending artery, *LCX* left circumflex artery, *RCA* right coronary artery, *TIMI* thrombolysis in myocardial infarction, *CK* creatine kinase, *AUC* area under the curve, *NT-proBNP* N-terminal pro brain natriuretic peptide, *eGFR* estimated glomerular filtration rate, *HbA1c* glycated hemoglobin

### LV structural characteristics

Left ventricular (LV) structural characteristics in the study population are presented in addition to reference values of healthy adults of comparable age (Table 2) [18]. LVESVi shows most deviations from reference values, followed by LVEDVi, and LVMi. LVESVi has the strongest correlation with infarct size as measured by CMR, followed by LVEDVi, and LVMi. EDWT and ESWT have no significant correlation with CMR determined infarct size.

**Table 2:** LV structural characteristics measured with CMR at 4 months

Study cohort n = 271 CMR parameter	(mean ± SD)	Correlation with infarct size (Pearson’s rho)	p	% above P95 or below P5
LVMi (g/m^2^)	50.4 ± 10.7	0.26	<0.001	17.2
LVEDVi (mL/m^2^)	96.1 ± 20.2	0.55	<0.001	46.4
LVESVi (mL/m^2^)	45.3 ± 17.1	0.70	<0.001	82.4
EDWT (mm)	5.91 ± 0.85	–	0.94	–
ESWT (mm)	9.36 ± 1.34	–	0.36	–
LVEF (%)	53.9 ± 8.5	−0.72	<0.001	79.4
Infarct size (% LVM)	9.0 ± 7.9	–	–	87.8

*LV* left ventricle, *CMR* cardiovascular magnetic resonance imaging, *SD* standard deviation, *P95* 95th percentile, *P5* 5th percentile, *SD* standard deviation, *LVMi* left ventricular mass index, *LVEDVi* left ventricular end diastolic volume index, *LVESVi* left ventricular end systolic volume index, *EDWT* end diastolic wall thickness, *ESWT* end systolic wall thickness, *LVEF* left ventricle ejection fraction

### Univariate analysis

Univariate linear regression results are presented in Tables 3 and 4. Randomization to metformin compared to placebo treatment had no effect on structural LV characteristics. Peak and AUC values for CK, CK-MB and Troponin T were significant predictors of LVEDVi, LVESVi and LVMi, but apart from AUC of Troponin T, did not significantly predict EDWT and ESWT. Strongest univariate predictors were peak Troponin T for LVEDVi, peak CK-MB for LVESVi, NT-proBNP at 2 weeks for LVMi, BSA for EDWT, and weight for ESWT.

**Table 3:** Univariate regression analysis of LV structural characteristics with clinical parameters

Factor	LVEDVi		LVESVi		LVMi		EDWT		ESWT	
	β	p	β	p	β	p	β	p	β	p
Age	−0.14	0.02	−0.06	0.29	0.00	0.96	0.12	0.052	0.12	0.053
Female sex	−0.13	0.03	−0.10	0.11	−0.24	<0.001	−0.28	<0.001	−0.23	<0.001
Body surface area	0.08	0.17	0.04	0.46	0.11	0.07	0.32	<0.001	0.28	<0.001
Weight	0.08	0.18	0.02	0.69	0.09	0.13	0.29	<0.001	0.29	<0.001
Height	0.12	0.047	0.08	0.17	0.17	0.005	0.26	<0.001	0.24	<0.001
Hypertension	−0.01	0.87	−0.01	0.82	0.13	0.04	0.20	0.002	0.14	0.03
Dyslipidemia	0.07	0.27	0.10	0.11	−0.04	0.47	−0.03	0.61	−0.05	0.40
Current smoking	−0.04	0.47	−0.03	0.58	0.04	0.49	0.04	0.51	0.04	0.53
Previous PCI	0.02	0.72	0.02	0.70	0.01	0.83	0.06	0.33	0.05	0.44
Systolic blood pressure	0.06	0.32	0.02	0.68	0.16	0.011	0.21	0.001	0.23	<0.001
Diastolic blood pressure	0.13	0.03	0.10	0.095	0.21	<0.001	0.19	0.003	0.23	<0.001
Heart rate	0.01	0.88	0.06	0.34	0.13	0.03	0.21	0.001	0.18	0.004
Ischemia time	0.00	0.97	0.04	0.50	0.04	0.56	−0.02	0.71	−0.01	0.89

*LV* left ventricle, *β* standardized beta, *LVEDVi* left ventricular end diastolic volume index, *LVESVi* left ventricular end systolic volume index, *LVMi* left ventricular mass index, *EDWT* end diastolic wall thickness, *ESWT* end systolic wall thickness

**Table 4:** Univariate regression analysis of LV structural characteristics with angiographic and biochemical parameters

Factor	LVEDVi		LVESVi		LVMi		EDWT		ESWT	
	β	p	β	p	β	p	β	p	β	p
Single vessel disease	0.00	0.97	−0.03	0.60	−0.03	0.67	0.01	0.89	0.01	0.89
Infarct-related artery		0.02		<0.001		0.06		0.003		0.26
LCX	−0.01	0.93	0.00	0.95	0.00	0.95	−0.10	0.15	−0.10	0.17
RCA	−0.17	0.01	−0.23	<0.001	−0.15	0.03	−0.23	0.001	−0.10	0.17
Infarct-related TIMI flow pre-PCI		<0.001		<0.001		0.02		0.33		0.57
TIMI 1	−0.02	0.74	−0.02	0.77	0.09	0.15	0.11	0.08	0.07	0.30
TIMI 2	−0.19	0.002	−0.19	0.002	−0.06	0.37	0.03	0.60	0.00	0.93
TIMI 3	−0.28	<0.001	−0.25	<0.001	−0.15	0.02	−0.01	0.86	−0.05	0.43
Infarct-related TIMI 3 post-PCI	−0.08	0.18	−0.09	0.12	−0.02	0.74	0.02	0.75	−0.03	0.60
Myocardial blush grade		0.08		0.03		0.31		0.99		0.70
MBG 1	0.08	0.52	0.08	0.52	0.05	0.72	−0.02	0.87	−0.13	0.32
MBG 2	−0.11	0.55	−0.09	0.64	−0.13	0.50	−0.04	0.84	−0.23	0.24
MBG 3	−0.13	0.52	−0.17	0.41	−0.09	0.66	−0.06	0.79	−0.22	0.29
Peak CK-MB	0.50	<0.001	0.64	<0.001	0.24	<0.001	0.07	0.25	−0.01	0.90
AUC CK-MB	0.49	<0.001	0.62	<0.001	0.26	<0.001	0.10	0.12	0.02	0.70
Peak CK	0.51	<0.001	0.64	<0.001	0.24	<0.001	0.07	0.14	0.00	0.97
AUC CK	0.51	<0.001	0.62	<0.001	0.26	<0.001	0.10	0.24	0.02	0.81
Peak Troponin T	0.51	<0.001	0.64	<0.001	0.27	<0.001	0.10	0.11	0.02	0.76
AUC Troponin T	0.51	<0.001	0.62	<0.001	0.32	<0.001	0.15	0.02	0.08	0.23
NT-proBNP	0.24	<0.001	0.23	<0.001	0.13	<0.001	0.20	0.001	0.20	0.002
NT-proBNP at 2 weeks	0.42	<0.001	0.49	<0.001	0.24	<0.001	0.27	<0.001	0.20	0.002
NT-proBNP at 6–8 weeks	0.51	<0.001	0.61	<0.001	0.19	<0.001	0.27	<0.001	0.18	0.007
eGFR	0.15	0.014	0.10	0.11	−0.01	0.89	−0.12	0.06	−0.10	0.10
Glucose	0.14	0.02	0.14	0.03	0.11	0.08	0.11	0.095	0.11	0.09
HbA1c	−0.08	0.21	−0.08	0.17	0.06	0.29	0.18	0.004	0.19	0.002

*LV* left ventricle, *β* standardized beta, *LVEDVi* left ventricular end diastolic volume index, *LVESVi* left ventricular end systolic volume index, *LVMi* left ventricular mass index, *EDWT* end diastolic wall thickness, *ESWT* end systolic wall thickness, *PCI* percutaneous coronary intervention, *LCX* left circumflex artery, *RCA* right coronary artery, *TIMI* thrombolysis in myocardial infarction, *CK* creatine kinase, *AUC* area under the curve, *NT-proBNP* N-terminal pro brain natriuretic peptide, *eGFR* estimated glomerular filtration rate, *HbA1c* glycated hemoglobin

### Multivariable analysis

Multivariable linear regression models of LVEDVi, LVESVi, LVMi, EDWT and ESWT are presented in Table 5. Forward and backward models were identical. Bootstrap models were identical, except the model for LVMi, which also included AUC Troponin T in the five most frequently selected variables, EDWT, which also included culprit in the right coronary artery (RCA) in the ten most frequently selected variables, and ESWT, which also included heart rate at admission in the nine most frequently selected variables. In the multivariable model for LVESVi, significant interactions were found for age with peak Troponin T and age with NT-proBNP at 5 months. Adding the interaction term between age and peak Troponin T increased the explained variance from 58 to 59%. In the multivariable model for LVEDVi, a significant interaction was found for age with sex, increasing explained variance from 49 to 50%. In the multivariable model for LVMi, significant interactions were found for NT-proBNP at 2 weeks with sex and hypertension at baseline. Adding the interaction term between NT-proBNP at 2 weeks and hypertension to the model increased the explained variance from 43 to 45%. In the multivariable model for EDWT, significant interactions were found for NT-proBNP at 2 weeks with sex, hypertension, heart rate and HbA1c. Adding the interaction term between NT-proBNP at 2 weeks and HbA1c increased the explained variance from 45 to 47% and dropped NT-proBNP at baseline out of the model. In the multivariable model for ESWT, significant interactions were found for NT-proBNP at 2 weeks with sex and HbA1c. Adding the interaction between NT-proBNP and HbA1c increased the explained variance from 35 to 37%, and dropped NT-proBNP at baseline, sex and current smoking out of the model.

**Table 5:** Multivariable regression analysis of LV structural characteristics

	Std. β	p-value
LVEDVi (R^2^ = 0.49)		
Age	−0.30	<0.001
Female sex	−0.16	0.001
Heart rate during CMR	−0.19	<0.001
AUC Troponin T	0.28	<0.001
NT-proBNP at 6–8 weeks	0.47	<0.001
LVESVi (R^2^ = 0.58)		
Age	−0.22	<0.001
Female sex	−0.14	0.001
Peak Troponin T	0.42	<0.001
NT-proBNP at 6–8 weeks	0.45	<0.001
LVMi (R^2 ^= 0.43)		
Age	−0.16	0.002
Female sex	−0.38	<0.001
Hypertension at baseline	0.16	0.008
Systolic blood pressure	0.16	0.003
NT-proBNP at 2 weeks	0.58	<0.001
EDWT (R^2^ = 0.45)		
Age	0.15	0.022
Female sex	−0.22	<0.001
Body surface area	0.41	<0.001
Heart rate at baseline	0.14	0.007
Heart rate during CMR	0.12	0.028
Hypertension in medical history	0.19	0.001
Current smoking	0.13	0.019
NT-proBNP at baseline	0.12	0.036
NT-proBNP at 2 weeks	0.32	<0.001
HbA1C	0.14	0.008
ESWT (R^2^ = 0.35)		
Age	0.21	<0.001
Female sex	−0.18	0.016
Body surface area	0.33	<0.001
Heart rate during CMR	0.14	0.011
Diastolic blood pressure	0.19	0.001
Current smoking	0.13	0.037
NT-proBNP at baseline	0.14	0.034
NT-proBNP at 2 weeks	0.21	0.002
HbA1c	0.16	0.006

*LV* left ventricle, *R²* coefficient of determination, *Std. β* standardized beta, *P95* 95th percentile of reference value, *LVMi* left ventricular mass index, *EDWT* end diastolic wall thickness, *ESWT* end systolic wall thickness, *LVEDVi* left ventricular end diastolic volume index, *LVESVi* left ventricular end systolic volume index, *NT-proBNP* N-terminal pro brain natriuretic peptide, *HbA1c* glycated hemoglobin, *TIMI* thrombolysis in myocardial infarction, *AUC* area under the curve, *CK* creatine kinase, *eGFR* estimated glomerular filtration rate

**Fig. 1:**
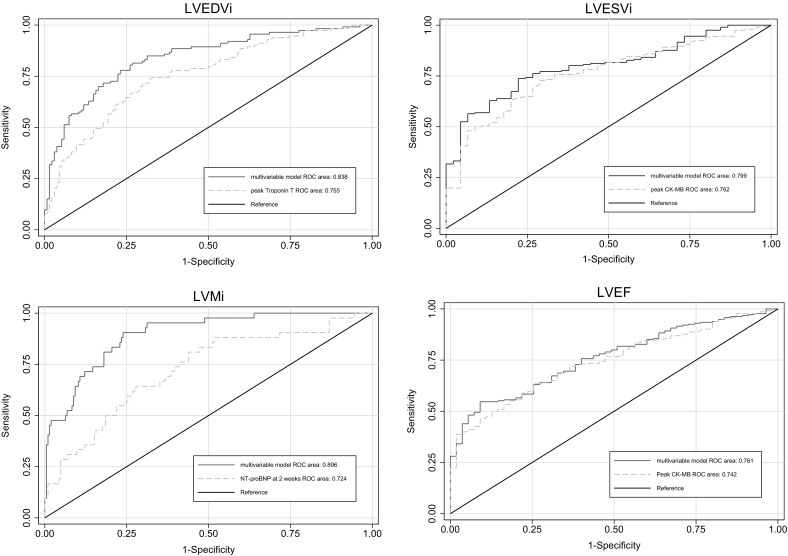
ROC curves of strongest univariate predictor and multivariable model predicting deviations from P95 reference values. *LVEDVi* left ventricular end diastolic volume index, *LVESVi* left ventricular end systolic volume index, *LVMi* left ventricular mass index, *ROC* receiver operating characteristic, *NT-proBNP* N-terminal pro brain natriuretic peptide, HbA1c glycated hemoglobin, *TIMI* Thrombolysis in Myocardial Infarction, *AUC* area under the curve, *CK* creatine kinase, *eGFR* estimated glomerular filtration rate, *CK* creatine kinase, *NT-proBNP* N-terminal pro brain natriuretic peptide

Delta values for NT-proBNP and eGFR between baseline and follow-up measures were tested in multivariable analysis but were not of additional value. Receiver operating characteristic (ROC) curves were made to analyze and visualize the performance of the multivariable models compared with the strongest univariate predictors in predicting LVEDVi, LVESVi and LVMi to be above P95 reference values (Fig. 1). In addition, a ROC curve was constructed for predicting deviations in LVEF, using peak CK-MB and the multivariable model described earlier [19]. The multivariable models for LVEDVi and LVMi had a significantly higher area under the ROC curve compared to the strongest univariate predictors (p = 0.004 and p < 0.001 respectively), the area under the ROC curve for LVESVi and LVEF did not improve (p = 0.11 and 0.29 respectively).

### Discussion

We studied the clinical, biochemical and angiographic predictors of LV remodeling characteristics 4 months after STEMI. Commonly used cardiac specific biomarkers for cardiac injury were the strongest predictors of the global structural LV remodeling response. We also considered the compensatory response of the remote non-infarcted LV wall thickness to STEMI. Interestingly, LV wall thickness of non-infarcted myocardium was not correlated with infarct size, biomarkers for cardiac injury, or other LV remodeling parameters, suggesting that the presumed compensatory response of remote myocardium is not related to cardiac injury caused by STEMI.

### Global remodeling

Left ventricular (LV) volume indices after STEMI are affected by adverse remodeling, as emphasized by the strong correlations with infarct size. Predictors of LV dilatation after STEMI in previous studies include infarct zone, wall motion score index, peak CK, extent of coronary artery disease, LV dyssynchrony, and early mitral regurgitation [7, 20, 21]. In daily clinical practice, physicians generally rely on enzymatic infarct size to predict LV remodeling. Our data suggests that NT-proBNP after 6–8 weeks is a better predictor of LV volume indices compared to (peak or AUC) Troponin T, CK, and CK-MB, and could be used more frequently in clinical practice for risk stratification and treatment optimization. Reference values for LV volume indices depend on age and sex, as reflected in both multivariable models [18, 22].

Within the multivariable model for LVEDVi, NT-proBNP was the strongest predictor, probably due to the relationship between LVEDV, wall stress and NT-proBNP. Heart rate during CMR assessment was an independent predictor of LVEDVi, which is a physiological effect resulting from increased LV filling times at lower heart rates.

LVESVi increases and LVEF declines in post-infarction LV remodeling [1]. In contrast with LVEDVi, the strongest predictor in the multivariable model for LVESVi was peak CK, emphasizing the strong relationship between infarct size and systolic function. Out of all remodeling parameters, LVESVi showed most deviations from reference values. The multivariable model had the highest explained variance and was comprised of biochemical biomarkers only.

LVMi was most strongly predicted by NT-proBNP, which was the only biochemical biomarker to remain significant in multivariable regression analysis adjusted for age, sex, hypertension, and blood pressure. NT-proBNP has been previously associated with LVM, and has been suggested to be used as a screening tool for LV hypertrophy [23–25]. NT-proBNP at 2 weeks predicts LVMi more accurately compared with levels at other time points suggesting, as might be expected, that NT-proBNP levels in the acute phase of STEMI are not representative. The predictive value of blood pressure for LVMi has been previously reported in healthy cohorts but is also a predictor at hospital admission for STEMI [26].

Previous studies in humans have shown that LVMi is reduced in the first months after infarction due to the loss of infarcted myocardium, although baseline measurements were always acquired after STEMI [27, 28]. We found a significant positive correlation of LVMi with infarct size as measured with CMR after 4 months. This could indicate that patients with LV hypertrophy develop a larger infarct size, which has been suggested before [29]. Alternatively, a compensatory hypertrophic response of the remaining, non-infarcted myocardium, as observed in previous animal studies, could explain the correlation between LVMi and infarct size [30, 31]. Biochemical biomarkers for myocardial injury were associated with LVMi, although this was not significant anymore after adjusting for NT-proBNP.

### Remote remodeling

We observed that predictors of LV wall thickness in the remote myocardium generally correspond with predictors of LVMi. In addition, HbA1c appeared an independent predictor of both increased EDWT and ESWT. In the GIPS-III trial, patients with known diabetes were excluded, but one out of six patients were diagnosed with diabetes after randomization [11]. In a previous study, patients with diabetes had higher LVMi, associated with an increased risk to develop HF after myocardial infarction [32]. This might be the result of a lower LV compliance. It remains speculative whether patients with high HbA1c levels had a higher pre-existing wall thickness or if this developed after STEMI. We also found that active smoking at baseline is an independent predictor of increased wall thickness, which has been described before in large cohorts [33].

A previous study reports patients after STEMI to have significantly higher wall thickness in non-infarcted myocardium, compared to control patients [27]. However, this was not adjusted for potential confounders such as hypertension. In our study, we found no correlation between infarct size and remote LV wall thickness. Also, levels of biochemical biomarkers for myocardial injury did not predict remote LV wall thickness, suggesting there might not be a compensatory hypertrophic response of remote non-infarcted myocardium at 4 months after STEMI.

### Limitations

There were several limitations to our study. We did not determine measures of myocardial edema, which might have influenced the relation of remote wall thickness with infarct size, and did not perform stress perfusion imaging to assess residual ischemia. CMR was performed at 4 months after STEMI, when LV remodeling might be ongoing. No additional CMR assessment was performed prior to, or after 4 months, to assess progression in remodeling. Mean infarct size (9% of LV mass) in our cohort was relatively small compared to other STEMI studies, possibly due to the inclusion criteria (first STEMI) and efficient management (median 155 min to treatment). Additional studies with a larger variability in infarct size might help to further investigate the relationship between remote myocardial remodeling after infarction. The use of in- and exclusion criteria of the GIPS-III trial has possibly affected our results. For example, known diabetes was an exclusion criteria and therefore the effect of prevalent diabetes on LV remodeling could not be studied.

## Conclusion

Our data underlines the strength of cardiac specific biomarkers in predicting global LV remodeling after STEMI. We found no evidence for the presumed hypertrophic response of remote non-infarcted myocardium at 4 months after STEMI.
